# Confirmation of the Reported Association of Clonal Chromosomal Mosaicism with an Increased Risk of Incident Hematologic Cancer

**DOI:** 10.1371/journal.pone.0059823

**Published:** 2013-03-22

**Authors:** Ursula M. Schick, Andrew McDavid, Paul K. Crane, Noah Weston, Kelly Ehrlich, Katherine M. Newton, Robert Wallace, Ebony Bookman, Tabitha Harrison, Aaron Aragaki, David R. Crosslin, Sophia S. Wang, Alex P. Reiner, Rebecca D. Jackson, Ulrike Peters, Eric B. Larson, Gail P. Jarvik, Christopher S. Carlson

**Affiliations:** 1 The Division of Public Health Sciences, Fred Hutchinson Cancer Research Center, Seattle, Washington, United States of America; 2 Department of Medicine, University of Washington, Seattle, Washington, United States of America; 3 Group Health Research Institute, Seattle, Washington, United States of America; 4 School of Public Health, University of Washington, Seattle, Washington, United States of America; 5 University of Iowa, College of Public Health, Iowa City, Iowa, United States of America; 6 National Human Genome Research Institute, National Institutes of Health, Bethesda, Maryland, United States of America; 7 Department of Genome Sciences, University of Washington, Seattle, Washington, United States of America; 8 Department of Medicine (Medical Genetics), University of Washington, Seattle, Washington, United States of America; 9 Division of Cancer Etiology, Department of Population Sciences, City of Hope and the Beckman Research Institute, Duarte, California, United States of America; 10 Department of Epidemiology, University of Washington, Seattle, Washington, United States of America; 11 Division of Endocrinology, Ohio State University, Columbus, Ohio, United States of America; University of Navarra, Center for Applied Medical Research, Spain

## Abstract

Chromosomal abnormalities provide clinical utility in the diagnosis and treatment of hematologic malignancies, and may be predictive of malignant transformation in individuals without apparent clinical presentation of a hematologic cancer. In an effort to confirm previous reports of an association between clonal mosaicism and incident hematologic cancer, we applied the anomDetectBAF algorithm to call chromosomal anomalies in genotype data from previously conducted Genome Wide Association Studies (GWAS). The genotypes were initially collected from DNA derived from peripheral blood of 12,176 participants in the Group Health electronic Medical Records and Genomics study (eMERGE) and the Women’s Health Initiative (WHI). We detected clonal mosaicism in 169 individuals (1.4%) and large clonal mosaic events (>2 mb) in 117 (1.0%) individuals. Though only 9.5% of clonal mosaic carriers had an incident diagnosis of hematologic cancer (multiple myeloma, myelodysplastic syndrome, lymphoma, or leukemia), the carriers had a 5.5-fold increased risk (95% CI: 3.3–9.3; p-value = 7.5×10^−11^) of developing these cancers subsequently. Carriers of large mosaic anomalies showed particularly pronounced risk of subsequent leukemia (HR = 19.2, 95% CI: 8.9–41.6; p-value = 7.3×10^−14^). Thus we independently confirm the association between detectable clonal mosaicism and hematologic cancer found previously in two recent publications.

## Introduction

Chromosomal mosaicism is the presence of differences in chromosomal content of cells within the same individual, derived from a single zygote [1]. The timing of the mutational event in development influences both the extent and types of mosaic cells (somatic and/or germinal) [2]. An early postzygotic mutation can result in chromosomal mosaicism in somatic cells, germinal cells, or occasionally both cell types [2], whereas events that occur later in life tend to be restricted to a particular cell lineage [3]. Chromosomal mosaicism contributes to inter-individual diversity and is an established cause of hypopigmentation of the skin [4], spontaneous abortions [5]–[7], birth defects [8], cognitive defects and brain development [9], [10], and various cancers [11]–[13] including hematologic cancers [3], [14], [15].

Hematologic cancers are a heterogeneous group of neoplastic conditions affecting the blood or blood-forming tissues. Observational data suggests that roughly half of patients with hematologic cancers harbor clonal chromosome abnormalities [15], which frequently fall at characteristic locations throughout the genome [3], [15]–[18]. Notably, chromosomal aneuploidy such 20q-, 13q-, 11q-, 17p-, 12+ and 8+ are commonly observed in affected individuals [3], [15]. Clinically, the identification of chromosomal aberrations is important in hematologic oncology for diagnosis, prognosis, treatment, and monitoring [19], [20].

Two recent population-based studies have identified an association between clonal chromosomal mosaicism (duplications, deletions, and copy neutral loss of heterozygosity) detected in genome-wide SNP array data and incident hematologic cancers [3], [15]. Employing the anomaly detection method previously described by Laurie et al. [3], we performed an independent investigation seeking to quantify the association between detectable chromosomal mosaicism and hematologic cancers in samples genotyped through the Electronic Medical Records and Genomics (eMERGE) network and the Women’s Health Initiative (WHI). Detectable mosaicism identified under this method requires a relatively high proportion of cells with the same abnormal karyotype (estimated at >5–10% abnormal cells [3]), thus we emphasize throughout the text that our study is limited to only the mosaicism we are capable of detecting. This work finds association between detectable clonal mosaicism and incident hematologic cancer, consistent with the previous studies.

## Results

Our study population of 12,176 individuals of predominantly European descent had previously been genotyped by the WHI and the eMERGE network for use in case-control studies of dementia, hip fractures, colorectal cancers, and metabolic and cardiovascular outcomes (**Table 1**). DNA samples were extracted from blood samples collected at or near to baseline for the primary study and were genotyped on an Illumina platform. Participant age at baseline ranged from 50–89 years, with a mean age across studies of 69 years (**Table 1** **, Figure S1**). Eligible participants needed to have high-quality genotype calls and be free of hematological cancer at baseline among other criteria, see Methods. The majority of subjects (60%) had over a decade of follow-up, with all eligible participants having at least a year of follow-up.

**Table 1:** Summary of characteristics of studies included in the analysis.

Study	n^1^	Illumina Genotyping Array(number of markers)	Initial Study Design (outcome)	Ancestry^2^	Mean Age^3^	Mean Follow-up(years)	DNA Source	Female (%)
**eMERGE (GHC/ACT)**	2357	Human660W_Quad_v1 (657,000)	Case-Control (Alzheimer's)	European	75.5	7.2	Blood	57.4
**WHI Hip Fracture** 4	4454	HumanHap550-2v3_B (555,000)	Case-Control (Hip Fractures)	European	68.9	11	Blood	100
**WHI GECCO** 5	858	HumanCytoSNP-12V2-1_A (220,00)	Case-Control (Colorectal Cancer)	European	65.1	11.8	Blood	100
**WHI GARNET**	4507	HumanOmni1-Quad_v1_0_B (1,000,000)	Case-Control (Metabolic and Cardiovascular)	European	65.3	11.1	Blood	100
**Total**	12176	N/A	N/A	European	68.6	10.4	Blood	91.8

1 Number of individuals included and analyzed in study;

2 Predominant ancestral group;

3 Age at baseline and/or sample collection;

4 Only phase 1 data included;

5 Only phase 2 data included.

Autosomal clonal chromosomal mosaicism was detected in 1.4% (n = 169) of the 12,176 individuals genotyped through the eMERGE and WHI studies. Mosaicism was more frequent among the 229 individuals with a qualifying incident hematologic cancer (case definition in **Table S1 in File S1)**, with 7.0% of cases harboring a detectable mosaic anomaly. Frequency of mosaic anomalies was observed to increase with age at sample collection, ranging from 0.9% for individuals less than 60 years of age at baseline to 2.7% for individuals above the age of 79 (**Figure S2**). There may also be suggestion of a relationship between age of collection and anomaly length (**Figure S3**).

**Figure 1:**
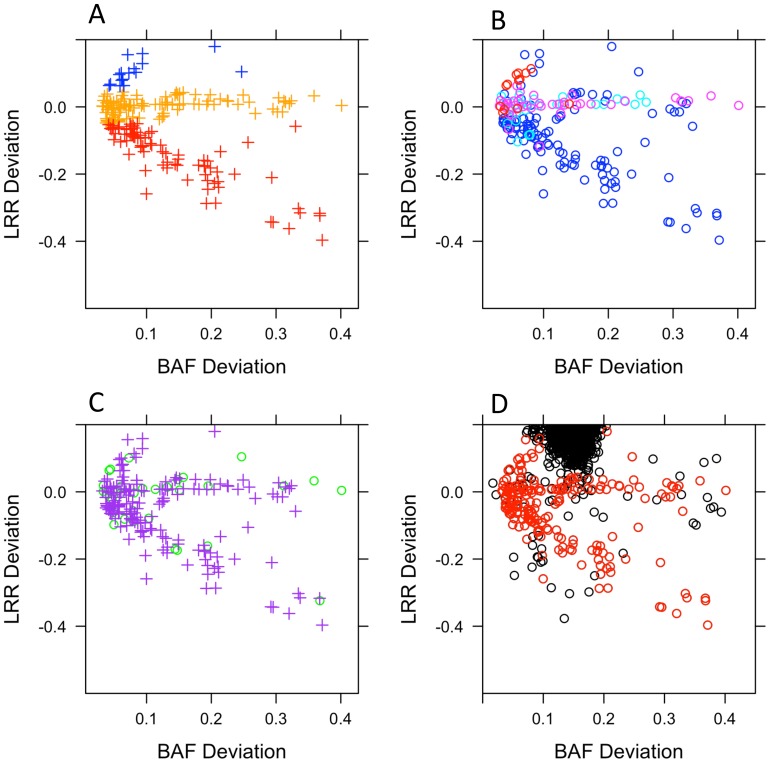
Characteristics of mosaic anomalies. A) BAF and LRR metrics for mosaic anomalies by estimated copy change from disomic state (red = loss, dark blue = gain, orange = copy neutral loss of heterozygosity. B) BAF and LRR metrics for mosaic anomalies by location (dark blue = interstitial, turquoise = p terminal, pink = q terminal or red = whole chromosome). C) BAF and LRR metrics for mosaic anomalies by type of chromosome (green circle = acrocentric, purple cross = metacentric). D) BAF and LRR metrics for mosaic (red) and non-mosaic (black) anomalies.

Most detected mosaic events were large (median size = 9.4 megabases, Mb). Of the mosaic anomalies detected in study subjects, 8.0% of anomalies were gains, 41.5% losses, and 50.5% copy neutral loss of heterozygosity (CN LOH) (**Table 2**). Median lengths of anomalies were 1.8 Mb for mosaic losses, 30.3 Mb for neutral events and 37.8 Mb for mosaic gains. Summary characteristics of the estimated copy change (gain, loss, CN LOH), chromosomal type (acrocentric, metacentric), chromosomal location, and distribution of mosaic to non-mosaic anomalies are plotted in **Figure 1** **A, B, C, & D,** respectively.

**Table 2:** Counts of detected mosaic anomalies by chromosomal location and event type.

Anomaly type	Event Type			
	Gain n	Loss n	CN LOH n	All anomalies n
Whole	8	0	5	13
p Terminal	0	3	26	29
q Terminal	1	2	35	38
Interstitial	7	78	35	120
All	16	83	101	200

**Figure 2:**
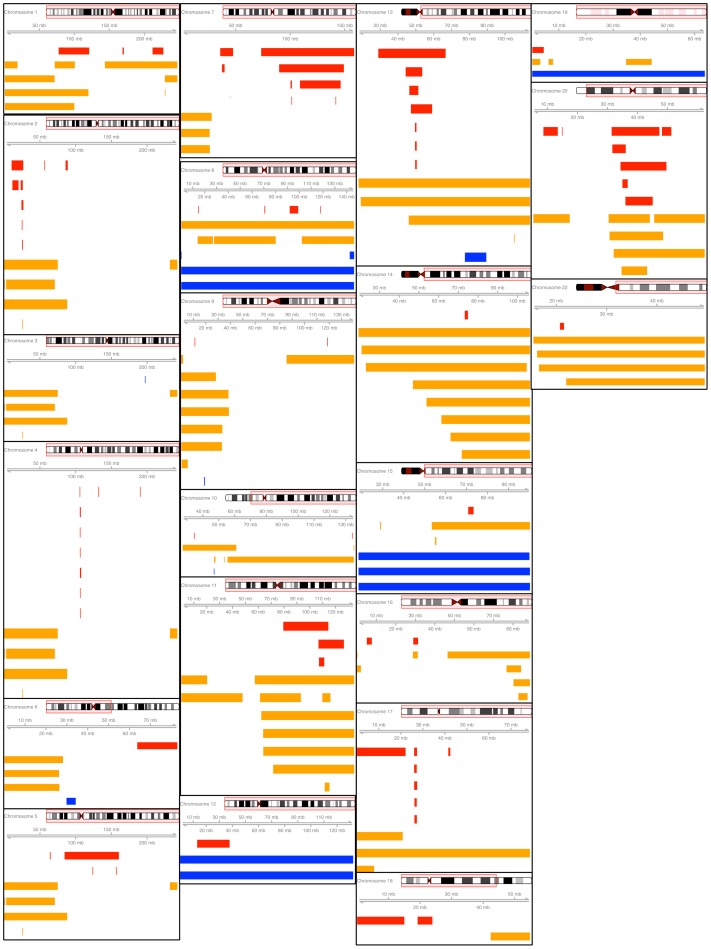
Mosaic anomalies plotted across chromosome in megabases (mb) by estimated copy change from disomic state (red = loss, dark blue = gain, orange = copy neutral loss of heterozygosity). The red box around the ideogram represents the region of interest for the plot located below. Chromosome 21 is omitted due to the absence of detected mosaic anomalies on the chromosome. (Note: plots are not drawn to scale).

Whole chromosome mosaic anomalies were detected on chromosomes 8 (1 CN LOH, 2 gain), 12 (2 gain), 13 (1 CN LOH), 14 (1 CN LOH), 15 (3 gain), 17 (1 CN LOH), 19 (1 gain) and 22 (1 CN LOH). Whole chromosomal mosaic anomalies represented only 6.5% of detected anomalies, whereas the majority of detected mosaic anomalies were either interstitial (60.0%) or terminal (33.5%). All mosaic events are shown graphically by chromosome in **Figure 2** and additional information on detected mosaic anomalies is provided in **Tables S2 & S3 in File S1**.

Recurrently deleted regions included 2p, 4q, 13q, 17q and 20q, which frequently overlapped with genes that have previously been associated with hematologic cancer (**Figure 2** **)**. On 2p there is a minimally deleted region of 751 kilobases (Kb) that is observed in 4 individuals. This region overlaps with the majority of the *DNMT3A,* a gene commonly mutated in T-cell lymphoma and myeloid leukemia [21]. Losses of 148 Kb on 4q, containing the *TET2* gene, were observed in 6 individuals. Loss of function mutations of the *TET2* gene are recurrently observed in myelodysplasia, myeloproliferative disorders and acute myeloid leukemia [22]. Of the 6 individuals with copy losses in the *TET2* gene region, 2 of these individuals had an incident hematologic cancer diagnosis (1 myelodysplastic syndrome & 1 multiple myeloma). Deletions of 13q were observed in 7 individuals with a minimally deleted region of 714 Kb that contains the *DLEU7* gene, which is thought to play a role as a tumor suppressor in chronic lymphocytic leukemia [23]. Mosaic deletions of *DLEU7* were observed 2 leukemia cases, 1 non-Hodgkin Lymphoma case and 4 individuals without a hematologic cancer. Mosaic deletions of 1 mb of 17q were observed in 5 hematologic cancer-free individuals. The recurrently deleted region on 17q overlaps with *NF1*, which is correlated with increased risk of pediatric leukemia [24]. Lastly, a repeatedly deleted region of 129 Kb on 20q was observed in 8 individuals. No candidate genes involved in hematologic malignancy were located in the minimally deleted region of 20q.

In total, 229 cases of incident hematologic cancer were observed in the 12,176 participants that did not have recorded hematologic cancer prior to enrollment or within 1 year of enrollment. Of the 229 cases, there were 51 leukemia, 6 Hodgkin lymphoma, 120 non-Hodgkin lymphoma, 46 multiple myeloma, and 6 myelodysplastic syndrome cases. Hematologic cancer cases had similar characteristics to individuals without detected hematologic cancer, however cases tended to be older at study baseline and tended to have shorter study follow-up **(** **Table 3** **)**. Using Cox proportional hazard models, we assessed the risk of incident hematologic cancer associated with the carriage of a mosaic anomaly to be 5.5 (95% CI: 3.3–9.3, p-value = 7.5×10^−11^) after adjustment for age at study intake and study cohort. Considering only incident hematologic cancers other than leukemia, risk estimates associated with mosaic anomalies were attenuated (HR = 3.2, 95% CI: 1.5–6.8, p-value = 0.003). Risk of leukemia associated with a mosaic anomaly was higher than the risk of other hematologic cancer with 9 of 51 leukemia cases with an identified anomaly versus 7 of 177 other hematologic cancer cases with an identified anomaly (p-value = 0.002).

**Table 3:** Comparison characteristics of hematologic cancer cases and individuals without a diagnosed hematologic cancer during study follow-up.

Phenotype	N	Age at Study enrollment mean(sd)	Years to diagnosis Median	Years of study follow-up Median	Mosaic n (%)	Non-Mosaic n (%)
**Leukemia**	51	71.2 (6.4)	4.9	8	9 (17.6)	42 (82.4)
**NHL**	120	70.83 (6.3)	5.5	10	5 (4.2)	115 (95.8)
**HL**	6	68.4 (3.0)	7.48	9	0 (0)	6 (100)
**MDS**	6	76.1 (6.7)	5	7.3	1 (16.7)	5 (83.3)
**MM**	46	70.8 (6.6)	4.9	8.1	1 (2.2)	45 (98.8)
**No Hematologic Cancer**	11947	68.5 (7.58)	NA	11.9	153 (1.3)	11794 (98.7)

**Figure 3:**
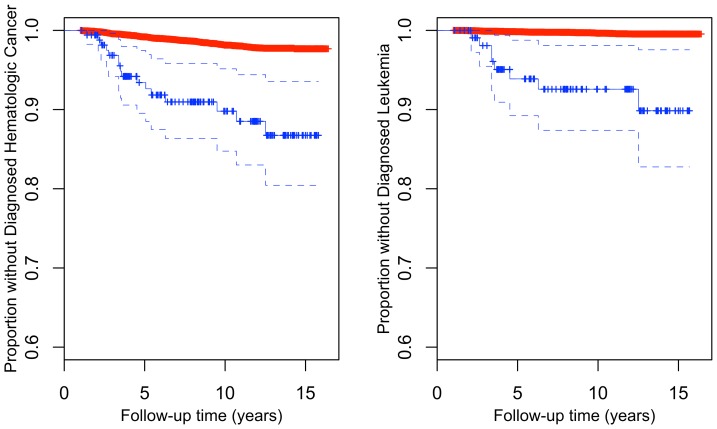
Kaplan Meier plots of the proportion of individuals remaining without diagnosed A) Hematologic cancer stratified by presence (blue) or absence (red) of a mosaic anomaly or B) Leukemia stratified by presence (blue) or absence (red) of a large mosaic anomaly (>2 mb).

Of the 16 hematologic cancer cases with detectable mosaicism, 9 of the cases were diagnosed with leukemia. Frequency of mosaicism among leukemia diagnosed cases was 17.6%, which is considerably higher than that observed for other hematologic cancers (3.9%) and much higher than rates observed in the putatively hematologic cancer-free sample (1.3%). Considering only leukemia as the outcome, the hazard ratio associated with a mosaic anomaly was 19.2 after adjustment for age at intake and cohort (95% CI: 8.9–41.6, p-value = 7.3×10^−14^). The association between large mosaic anomalies and incident hematologic outcomes other than leukemia is much attenuated, if it holds at all (HR = 2.7, 95% CI: 0.98–7.2, p-value = 0.056). For all hematologic cancers and specifically for leukemia, likelihood of remaining undiagnosed differed between mosaic and non-mosaic carriers (**Figure 3A & B** **, respectively**).

## Discussion

We confirm the association between chromosomal mosaicism detected in blood leukocyte DNA and a diagnosis of hematologic cancer in the following decade. A previous report by Laurie et al. [3] estimated the risk of hematologic malignancy associated with a mosaic anomaly to be 10-fold greater than the risk experienced by individuals without a detected anomaly. Using a population with considerably more hematologic cancer cases, our estimate of a 5.4-fold increased risk is confirmatory of a strong association between mosaic anomalies and hematologic cancer. Jacobs et al. [15] reported an estimated 35-fold increased risk of subsequent leukemia diagnosis associated with carriage of a large detectable mosaic anomaly (>2 Mb). We found the risk of leukemia associated with a large mosaic anomaly to be 19.2-fold higher than the risk of non-mosaic individuals. Though our risk estimates are somewhat smaller compared to the previous findings, these results provide strong, independent confirmation of the reported results (**Table 4**). Similarities between detected mosaic anomalies and recurrent anomalies previously observed in hematologic cancer (deletions involving the regions 2p-, 4q-, 13q-, 17q- and 20q-) provide additional support for the association.

**Table 4:** Comparison of previously reported hazard ratios to results from this study.

Study	Association	Incident HematologicCancer Cases (n)	HR [95% CI]	P-value
Schick et al.	Hematologic cancer ∼ detected mosaic anomaly	229	5.5 [3.3–9.3]*	7.5×10^−11^
Laurie et al.	Hematologic cancer ∼ detected mosaic anomaly	105	10.1 [5.8–17.7]	3.0×10^−10^
Schick et al.	Leukemia ∼ large detected mosaic anomaly (>2 Mb)	51	19.2 [8.9–40.6]*	7.3×10^−14^
Jacobs et al.	Leukemia ∼ large detected mosaic anomaly (>2 Mb)	43	35.4 [14.7–76.6]	3.8×10^−11^

* Referent: no detected mosaic anomalies.

In our analysis of middle-aged to older adults (mean 69 years; range 50–89), we observed increasing frequency of detectable mosaicism with increasing age. This result confirms findings by the two prior GWAS-based mosaicism studies [3], [15] that report that frequency of mosaic events are low in individuals younger than 50 years (0.23–0.5%), but rise about 2–3% in individuals in their mid-70s. These observations may be related to age-related somatic mutation accumulation or declines in efficiency of DNA repair mechanisms [25].

Our confirmation study includes as many incident hematologic cancer cases as the two previous studies combined (229 cases vs. 105 [3] and 115 cases [15]). The association between detected chromosomal mosaicism and hematologic cancer we observe is both significant statistically and substantial in magnitude, suggesting this finding is robust across studies. However, in this and other studies the cancer type with the strongest association has been leukemia, with the association among the other hematologic cancers weaker, if present at all. Assessment of additional non-leukemia hematologic cancers in a well-powered comparison would help clarify the existence and magnitude of a non-leukemia association. Additional characterization of the association between other somatic changes including balanced transversions and translocations that cannot be detected using this method could be a useful extension of this work.

There also remain questions about the detection of karyotypic abnormalities from blood-derived DNA. Longitudinal genotyping studies or comparative genomic hybridization could explore the trajectory of clonal expansion among individuals with mosaic anomalies. It would be of interest to determine whether these trajectories differ between subjects who developed clinically diagnosed cancers and those who did not. In individuals with putative mosaicism, it would also be of interest to test other tissues for the presence of the mosaic anomaly. Although we conjecture that bona fide mosaicism would be typically confined to the leukocytes, studies to date have been unable to test this hypothesis as they have lacked access to other tissues from affected individuals.

In summary, our study provides confirmation of the Laurie et al. [3] and Jacobs et al. [15] studies. These three studies a report a sizeable risk of hematologic cancer associated with detected mosaic anomalies in blood, however the mechanisms underlying this association are unclear. Though tumorigenesis is often accompanied by chromosome instability and alteration of chromosome copy number, the molecular mechanisms responsible for chromosomal mosaicism and the inter-relationships between chromosomal mosaicism and hematologic cancers remain poorly understood. Tumor suppressor proteins and genes that control proper centrosome formation, mitotic checkpoint signaling, and movement of duplicated chromosomes may be involved in both phenomena [26]. In childhood leukemias, chromosomal anomalies and preleukemic clones appear to arise prenatally, suggesting that secondary genetic events that occur postnatally are required for the development of disease [14]. In adult hematologic cancers, it is possible that multiple pathogenic (genetic and environmental) events over time, together with an accumulation of somatic mutations, promotes tumorigenesis, and ultimately the occurrence of overt disease. Further study is warranted to identify and characterize mechanisms underlying this association.

## Materials and Methods

### eMERGE Study Population

The eMERGE sample was recruited from the Group Health Cooperative (GHC) health maintenance organization in the Seattle-Puget Sound area. Study participants enrolled into either the University of Washington/Group Health Cooperative Alzheimer’s Disease Patient Registry (ADPR) [27] or the Adult Changes in Thought study (ACT) [28]. Both ADPR and ACT were initially proposed to study dementia. The ADPR was an incident case finding study of dementia that began in 1986 to both demonstrate feasibility of registries for research and seek markers for Alzheimer’s disease and related dementias. The ACT study began in 1994 as a planned successor to the ADPR. The ACT study initially enrolled 2,581 subjects of primarily European descent without dementia among individuals over the age of 64 years. The predominance of individuals of European descent is a feature of the population of the Puget Sound region, rather than a selection criterion. ACT has since evolved to a community-based cohort study of approximately 2,000 cognitively intact individuals through continuous enrollment. A set of 2,357 unrelated participants aged 50–89 years were genotyped using DNA derived from peripheral blood samples as part of the eMERGE project, and these individuals were included in our present study. Both cases and controls from the dementia study were included in the study population (**Table S4 in File S1, Table S5 in File S1**).

Clinical follow-up data, in the form of comprehensive clinical medical, laboratory and pharmacy records, are available for all ACT and ADPR participants, with a median of 6.1 years of electronic medical record follow-up after study enrollment. Subjects consented to genotyping as a part of the eMERGE study and approval for these particular analysis were obtained from the Group Health Cooperative Institutional Review Board.

### WHI Study Population

The Women’s Health Initiative (WHI) is a large cohort study with a primary focus on cardiovascular disease and breast cancer, but with secondary outcomes that include several adjudicated hematologic cancers [29]. WHI participants with Illumina GWAS data were available from three separate case-control GWAS studies within WHI: a study of colorectal cancer (GECCO; Peters, PI), a study of hip fractures (Hip Fracture; Jackson, PI), and a study of hormone treatment and cardiovascular disease/metabolic outcomes (GARNET; Reiner, PI). WHI approval was obtained for the inclusion of these samples in this study. In total, 9,819 previously genotyped WHI participants of primarily European descent were included in the analysis. Again, descent was not a selection criterion, but rather relates to the underlying population demographics. WHI participants were female, aged 50–79 years at enrollment, consented for research on age-related health issues, with an average of 12 years follow-up after recruitment. We observed no correlation between case status and either detected anomalies or subsequent hematologic cancer, and, therefore, included all eligible cases and controls in our analyses (**Table S4 in File S1**).

### Classification of Clinical Outcomes of Interest and Relevant Covariates

The primary clinical outcomes of interest in this study were hematologic cancers, including leukemia, lymphoma, myelodysplastic syndrome and multiple myeloma. Clinical outcomes within the eMERGE sample were ascertained through electronic medical reporting of International Classification of Diseases, ninth revision (ICD-9) codes 200.0–208.9, 238.6, 238.72–238.75 and 238.79 **(Table S1 in File S1**). ICD-9 diagnosis classification for hematologic cancers was obtained from the Washington State Cancer Registry definitions [30] with physician classification of myelodysplastic syndrome ICD-9 coding.

Adjudicated clinical outcomes during WHI follow-up included leukemia, Hodgkin and non-Hodgkin lymphoma, and multiple myeloma. Ascertainment of clinical outcomes occurred according to previously described WHI methods [29]. Briefly, hematologic cancer outcomes were ascertained by self-report at annual or bi-annual follow-up with participants, with subsequent physician adjudication based on medical records and pathology reports to confirm hematologic cancer cases and assign an International Classification of Disease for Oncology, 3^rd^ edition code when appropriate. With the exception of multiple myeloma, self-reported history of hematologic cancer prior to WHI enrollment was available for participants.

We considered participants to be putatively hematologic cancer-free at baseline if they reported no history of a hematologic cancer at intake, and had no hematologic cancer diagnosis within the first year of study follow-up. Participants reporting a cancer history, early diagnosis, missing history, or less than a year of follow-up were excluded from all analyses and subject counts.

### Genotyping and Quality Control

The eMERGE samples were genotyped on the Illumina Human 660W-Quadv1_A array at the Center for Inherited Diseases Research at Johns Hopkins University. WHI samples were genotyped on the following Illumina platforms: HumanOmni1_Quad_v1_0_B, Human HapMap 550K and HUMAN CYTOSNP-12 at the Broad Institute of MIT and Harvard, and the Translational Genomics Research Institute **(** **Table 1** **)**. B-Allele Frequency (BAF) and log R ratio (LRR) metrics were calculated using the Illumina BeadStudio software.

Standard quality control was conducted to eliminate samples of unsure identity or DNA quality [31]. We screened for sex discordant samples using estimates of X chromosome heterozygosity, and unintentional duplicates (genotyping controls and samples duplicated across studies) through a global estimate of the proportion identity by state [32]. We excluded samples from analysis if genotype call rates were less than 98% or if the BAF standard deviation of non-anomalous autosomal chromosomes exceeded 0.06. In total, 723 samples were excluded from the analysis. Of the excluded samples, 23 samples were excluded for high non-anomalous BAF standard deviation of autosomal chromosomes, 610 for unintentional duplicates, 86 for low genotyping call rate, and 4 for gender-mismatch. Most of the unintentional duplicates are attributable to substantial overlap between cohorts genotyped in WHI.

### Anomaly Detection and Quality Control

Chromosomal anomalies were detected through the “anomDetectBAF” method and all quality control was carried out using the “anomIdentifyLowQuality” method from the GWASTools package version 1.2.1 available for R version 2.15 through the Bioconductor repository [33]. A detailed description of these methods can be found in Laurie et al. [3].

The “anomDetectBAF” method is capable of detecting copy gain and loss for segments of length greater than 50 kb in Illumina high-density SNP genotyping data, as well as mosaic CN LOH. In brief, the “anomDetectBAF” detection method relies on circular binary segmentation [34] to segment chromosomes based on change-points in the relative allelic intensity (B-allele frequency; BAF). On each chromosome, heterozygous and missing SNPs genotypes are identified, and the BAF at these loci is transformed (tBAF: sqrt(min(BAF,1-BAF,abs(BAF-median BAF))). Anomalous segments are called based on deviation from non-anomalous baseline. This implies that the change-points estimated have uncertainties that depend on the density of SNPs surrounding the anomaly that are heterozygous in the individual of question. A conservative estimate of the endpoint uncertainty would be on the order of 10–50 kilobases typically. Only anomalies on the autosomes were included in the analyses due to the inherent copy-number difference between males and females.

Following anomaly detection, the quality control pipeline detailed in Laurie et al. [3] was utilized to filter false positive anomalies. The quality control pipeline used both bioinformatic approaches and manual review, consisting of visual screening of detected anomalies through LRR and BAF plots, to identify false positive and improperly segmented anomalies. Anomalies were screened with the “anomIdentifyLowQuality” using the suggested parameters for BAF anomalies (sd.thresh = 0.1, sng.seg.thresh = 0.0008, auto.seg.thresh = 0.0001). Low quality BAF anomalies were those that had high variance (BAF or LRR standard deviation exceeding 0.1) or were highly segmented (number of segments/number of eligible SNP>0.0001 for an autosome). Low quality samples were considered ineligible and were excluded from the analysis. Using manual review, adjacent anomalies (<300 probes between anomalies) with similar LRR values were merged and breakpoints of incorrectly segmented anomalies were adjusted. Anomalies spanning the centromere were screened using manual review with the criteria that BAF anomalies have at least 500 probes on each side of the centromere and breakpoints were adjusted, as needed from chromosomes failing the fit the criteria for a centromere-spanning anomaly. Anomalies were required to have at least 50 BAF eligible probes to be included in the study. Manual review was conducted on all anomalies larger than 2 Mb and all anomalies classified as mosaic.

We classified anomalies as constitutive (potentially germline CNV) and mosaic (acquired somatic) based on the observation that constitutive anomalies primarily fall within the 3N (trisomic) space [3]. In order to define mosaic anomalies, we plotted per subject-chromosome LRR deviation (median(anomalous LRR)- median(nonanomalous LRR)) vs. BAF median absolute deviation (MAD, Median|anomalous BAF-Median(nonanomalous BAF)|), and then used k-means clustering (k = 3) to distinguish between the large constitutive centroid and the putatively mosaic anomalies.

We defined constitutive anomalies as those within two MADs from the median of the cluster. Manual review of putatively mosaic anomalies was used to distinguish between mosaic anomalies and technical artifacts (improperly segmented or false positive anomalies). Anomalies defined as constitutive or artifacts were excluded and therefore not considered in further analyses. Consistent with Laurie et al. [3], we only attempted to detect mosaics in which one of the mixing populations was diploid. Clustering of constitutive anomalies is displayed in **Figure 1D**.

Mosaic anomalies were classified as copy neural loss of heterozygosity, loss, or gain based on LRR and BAF deviation metrics (**Figure 1A**). Anomalies with an LRR deviation (|anomalous LRR- non-anomalous LRR|) of less than 0.05 were classified as copy neutral loss of heterozygosity. Anomalies with an LRR deviation greater than 0.05 or less than −0.05 were classified as gains or losses, respectively. Manual review was used to confirm classification in ambiguous cases.

### Statistical Analysis

All statistical analyses were carried out in R version 2.15.0 [35]. Anomaly calling was carried out using the “anomDetectBAF” wrapper of the “anomSegmentBAF” and “anomFilterBAF” functions located in the GWASTools package. Low quality samples were removed using the “anomIdentifyLowQuality” in the GWASTools package. We utilized the “coxph” function with the “survival” package for Cox proportional hazard ratio estimates and to fit Kaplan Meier curves, and the “kmeans” function of the “stats” package to cluster putative constitutive anomalies.

We evaluated the association between detectable mosaic chromosomal anomalies with incident hematological cancer through modeling of Cox proportional Hazard Ratios, 95% Confidence Intervals and p-values. For consistency with previous reports, we report hazard ratios for the association between mosaic anomalies and all hematologic cancers and hazard ratios for the association between large mosaic anomalies (>2 Mb) and incident leukemia. For all Cox proportional hazard ratio estimates, we used the robust variance option. Survival time was assessed as time between specimen collection or intake and hematologic cancer diagnosis for individuals with cancer and as time between specimen collection or intake and death or study attrition for unaffected individuals. Incident hematologic cancer diagnosis was considered as the event indicator. To ensure that our estimates were robust to the influence of other confounding factors, we performed multivariate analysis including age, sex, ethnicity and initial study as covariates. In final estimates, we adjusted for age at specimen collection or intake based on the observation that frequency of anomalies generally increases with age. Age is also correlated with frequency of hematologic cancer with the median age of onset between 65–70 years [36]. Estimates were also adjusted for initial study cohort. The reference group for hazard estimates was composed of individuals without a detected mosaic anomaly.

## Supporting Information

Figure S1 **Distribution of age at baseline across studies.** The red dotted line represents the median age of the study.
(TIF)

Figure S2 **Percent of individuals with a mosaic anomaly across 5-year age bins.**
(TIF)

Figure S3 **Distribution of size of mosaic anomaly versus age of specimen collection (or intake surrogate) by estimated copy change from disomic state (red = loss, dark blue = gain, orange = copy neutral loss of heterozygosity).**
(TIF)

File S1 **Supplementary tables.**
(DOCX)
