# Supplementary figures and images for: Confirmation of the Reported Association of Clonal Chromosomal Mosaicism with an Increased Risk of Incident Hematologic Cancer
Source: PLoS One. 2013 Mar 22;8(3):e59823. doi: 10.1371/journal.pone.0059823 (PMC3606281; doi:10.1371/journal.pone.0059823)

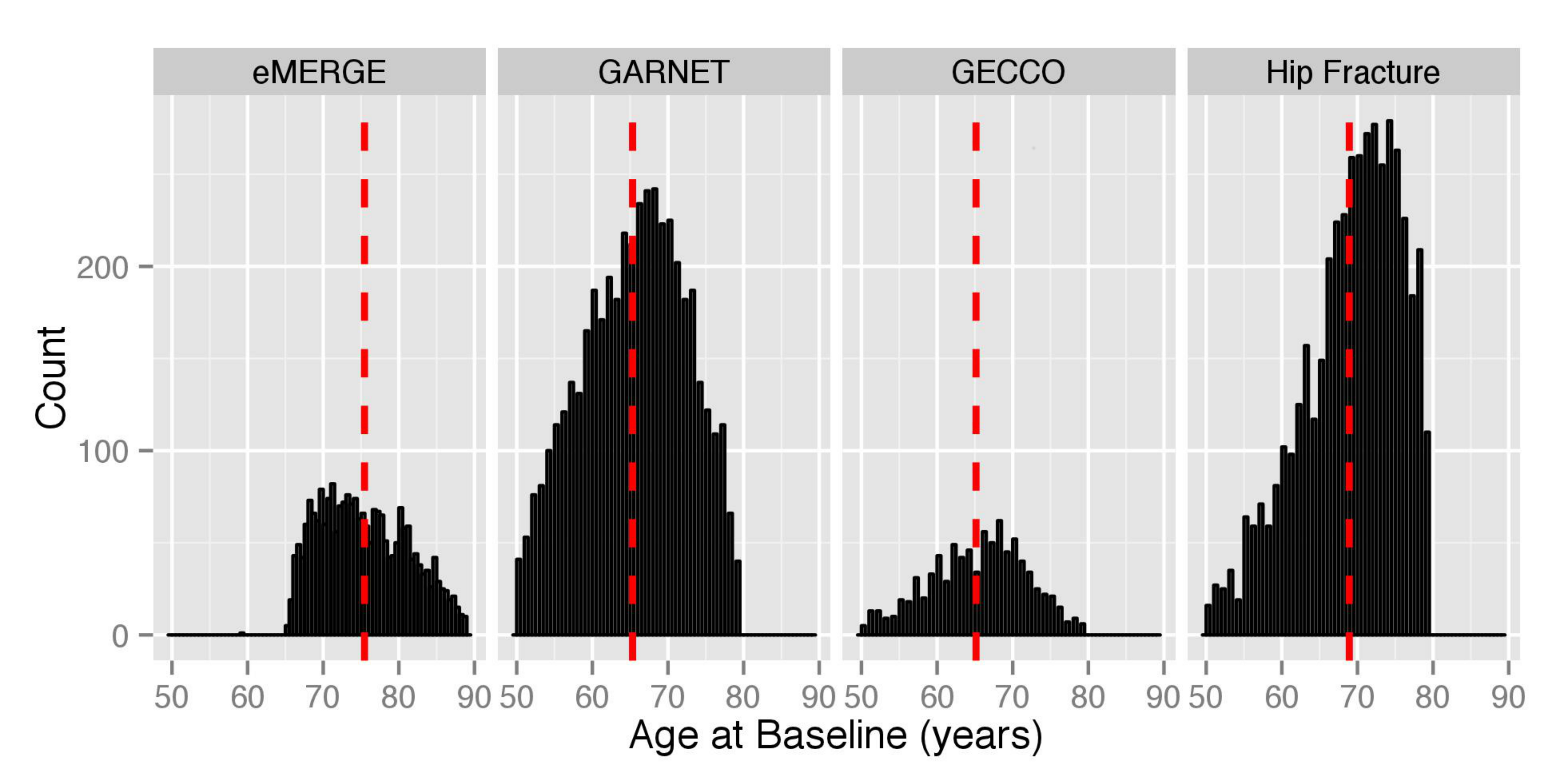

Supplement: Figure S1 — Distribution of age at baseline across studies. The red dotted line represents the median age of the study. (TIF) [file pone.0059823.s001.tif]

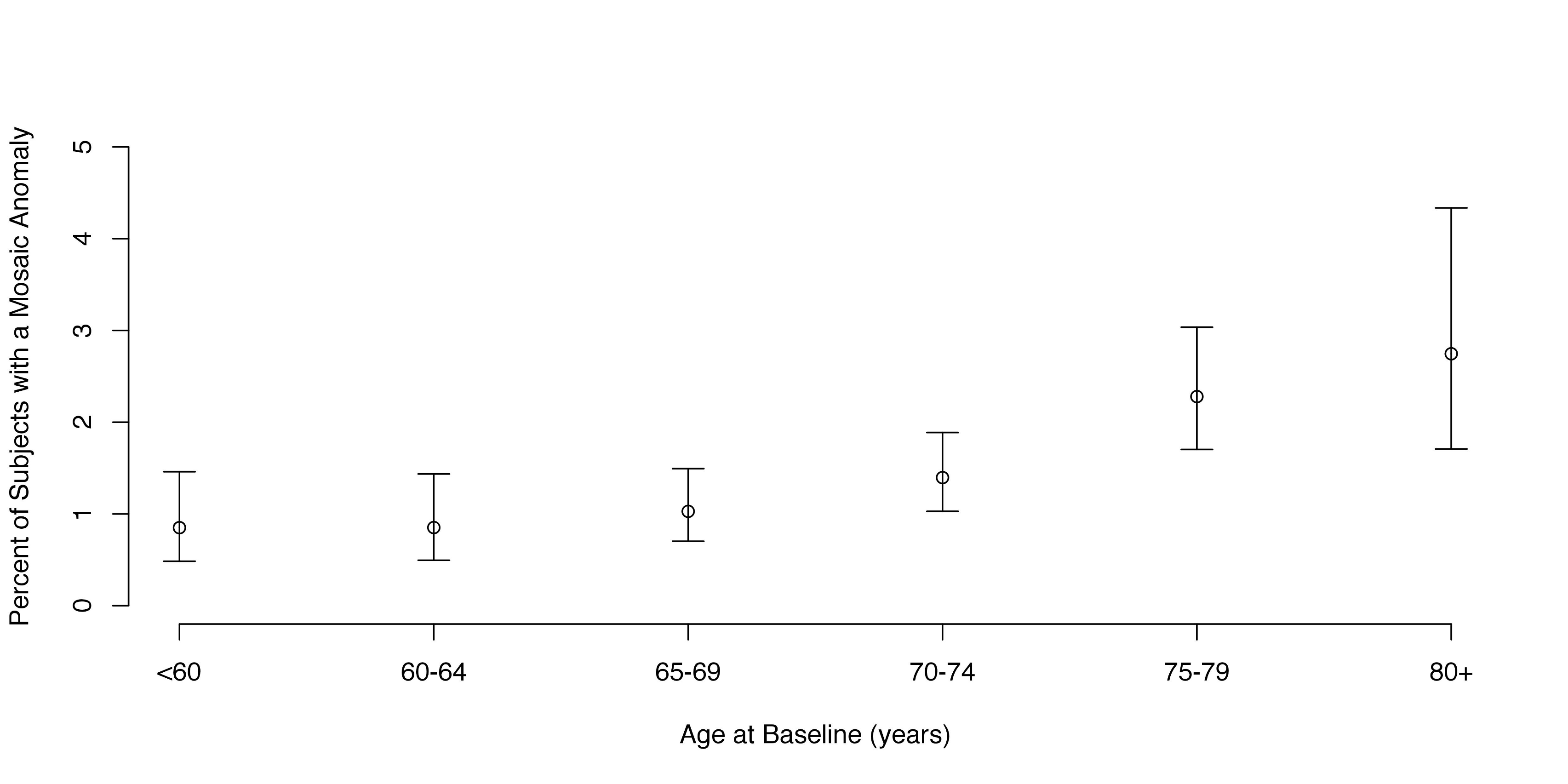

Supplement: Figure S2 — Percent of individuals with a mosaic anomaly across 5-year age bins. (TIF) [file pone.0059823.s002.tif]

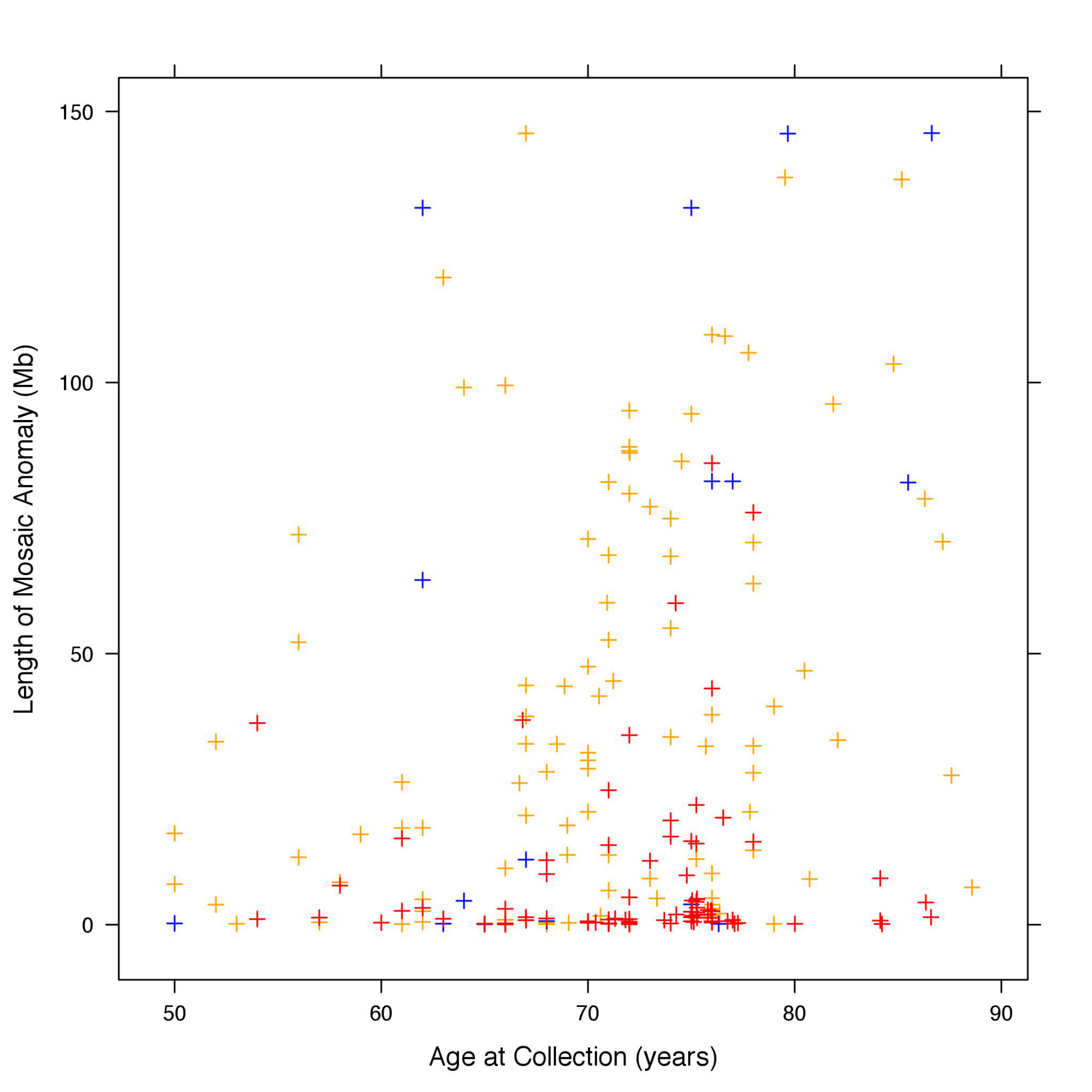

Supplement: Figure S3 — Distribution of size of mosaic anomaly versus age of specimen collection (or intake surrogate) by estimated copy change from disomic state (red = loss, dark blue = gain, orange = copy neutral loss of heterozygosity). (TIF) [file pone.0059823.s003.tif]
