# Supplementary material for: Confirmation of the Reported Association of Clonal Chromosomal Mosaicism with an Increased Risk of Incident Hematologic Cancer
Source: PLoS One. 2013 Mar 22;8(3):e59823. doi: 10.1371/journal.pone.0059823 (PMC3606281; doi:10.1371/journal.pone.0059823)
Supplement: File S1 — Supplementary tables. (DOCX) [file pone.0059823.s004.docx]

**Supplementary Tables**

**Table S1.** Classification of hematologic cancer by ICD-9 and ICD-O3 medical billing codes in eMERGE and WHI.

| **Hematologic Diagnosis** | **eMERGE (ICD-9)** | **WHI (ICD-O3 Histology Code)** |
| --- | --- | --- |
| Hodgkin Lymphoma | 201.0-201.9 | 9650, 9663, 9680 |
| Non-Hodgkin Lymphoma | 200.0-200.8, 202.0-202.2, 202.8-202.9 | 9590, 9591, 9670, 9671, 9673, 9680, 9684, 9687, 9689, 9690, 9691, 9695, 9698, 9699, 9702, 9705, 9714, 9729 |
| Leukemia | 204.0-208.9 | 9823,9834,9836,9840,9861,9874,9875,9895, 9940,9945 |
| Multiple Myeloma | 203, 238.6 | 9731, 9732 |
| Myelodysplastic Syndrome | 238.72-238.75, 238.79 | NA |

**Table S2.** Counts by type of mosaic anomalies across chromosomes by cancer status.

| **No Incident Hematologic Cancer** | | | | | | | | | | | | | | | | | | | | | |
| --- | --- | --- | --- | --- | --- | --- | --- | --- | --- | --- | --- | --- | --- | --- | --- | --- | --- | --- | --- | --- | --- |
|  | **Chromosome** | | | | | | | | | | | | | | | | | | | | |
| **Anomaly type** | **1** | **2** | **3** | **4** | **5** | **6** | **7** | **8** | **9** | **10** | **11** | **12** | **13** | **14** | **15** | **16** | **17** | **18** | **19** | **20** | **22** |
| interstitial | 4 | 9 | 9 | 9 | 2 | 2 | 10 | 7 | 3 | 8 | 4 | 1 | 6 | 1 | 3 | 6 | 6 | 2 | 3 | 11 | 1 |
| p terminal | 4 | 4 | 0 | 2 | 0 | 2 | 0 | 0 | 6 | 0 | 2 | 0 | 0 | 0 | 0 | 1 | 2 | 0 | 1 | 0 | 0 |
| q terminal | 2 | 1 | 0 | 6 | 0 | 0 | 1 | 2 | 1 | 0 | 6 | 0 | 2 | 6 | 1 | 2 | 0 | 0 | 0 | 2 | 3 |
| whole | 0 | 0 | 0 | 0 | 0 | 0 | 0 | 2 | 0 | 0 | 0 | 2 | 0 | 1 | 3 | 0 | 1 | 0 | 1 | 0 | 1 |
| **Incident Hematologic Cancer** | | | | | | | | | | | | | | | | | | | | | |
|  | **Chromosome** | | | | | | | | | | | | | | | | | | | | |
| **Anomaly Type** |  |  |  |  |  |  |  |  |  |  |  |  |  |  |  |  |  |  |  |  |  |
| interstitial | 1 | 0 | 0 | 2 | 3 | 0 | 2 | 0 | 0 | 0 | 1 | 0 | 3 | 0 | 0 | 0 | 0 | 0 | 0 | 1 | 0 |
| p terminal | 0 | 0 | 0 | 0 | 0 | 1 | 0 | 0 | 1 | 0 | 0 | 0 | 0 | 0 | 0 | 0 | 1 | 1 | 1 | 0 | 0 |
| q terminal | 0 | 0 | 0 | 1 | 0 | 0 | 1 | 0 | 0 | 0 | 0 | 0 | 0 | 1 | 0 | 0 | 0 | 0 | 0 | 0 | 0 |
| whole | 0 | 0 | 0 | 0 | 0 | 0 | 0 | 1 | 0 | 0 | 0 | 0 | 1 | 0 | 0 | 0 | 0 | 0 | 0 | 0 | 0 |

**Table S3.** Detailed information about all detected mosaic anomalies. Anomaly starting and ending bases are hg18 coordinates of flanking heterogeneous probes called as anomalous by algorithm, but note will endpoints have uncertainties on the order of 10-50 kilobases.

| Individual Identifier | Cohort | Chromo-some | Sex | Anomaly Starting Base | Anomaly Ending Base | Anomaly Length | Cancer Status | Diagnostic Category |
| --- | --- | --- | --- | --- | --- | --- | --- | --- |
| 115 | emerge | 16 | male | 5124912 | 7694747 | 2569836 | 0 | NA |
| 116 | emerge | 14 | male | 73266439 | 74888300 | 1621862 | 0 | NA |
| 116 | emerge | 20 | male | 31603948 | 36060424 | 4456477 | 0 | NA |
| 117 | emerge | 4 | male | 85676137 | 191164126 | 105487990 | 0 | NA |
| 118 | emerge | 16 | male | 80252351 | 88677423 | 8425073 | 0 | NA |
| 119 | emerge | 4 | female | 82596353 | 191164126 | 108567774 | 0 | NA |
| 120 | emerge | 7 | male | 101378758 | 101558266 | 179509 | 0 | NA |
| 121 | emerge | 17 | female | 51088 | 22133072 | 22081985 | 1 | LEUKEMIA |
| 121 | emerge | 18 | female | 102535 | 15057124 | 14954590 | 1 | LEUKEMIA |
| 121 | emerge | 19 | female | 243082 | 4461560 | 4218479 | 1 | LEUKEMIA |
| 121 | emerge | 20 | female | 5307457 | 17405674 | 12098218 | 1 | LEUKEMIA |
| 122 | emerge | 1 | female | 143666208 | 247093596 | 103427389 | 0 | NA |
| 123 | emerge | 14 | male | 61417262 | 106358708 | 44941447 | 1 | LEUKEMIA |
| 124 | emerge | 2 | female | 11444167 | 19994560 | 8550394 | 0 | NA |
| 124 | emerge | 2 | female | 24653351 | 25404634 | 751284 | 0 | NA |
| 125 | emerge | 4 | female | 132116590 | 132525024 | 408435 | 0 | NA |
| 126 | emerge | 1 | male | 168882541 | 170720463 | 1837923 | 0 | NA |
| 126 | emerge | 3 | male | 70689611 | 72954463 | 2264853 | 0 | NA |
| 126 | emerge | 3 | male | 163214006 | 164488710 | 1274705 | 0 | NA |
| 126 | emerge | 18 | male | 19250399 | 23936078 | 4685680 | 0 | NA |
| 126 | emerge | 20 | male | 8652552 | 13439522 | 4786971 | 0 | NA |
| 126 | emerge | 20 | male | 48108756 | 51242306 | 3133551 | 0 | NA |
| 126 | emerge | 20 | male | 34952889 | 36718407 | 1765519 | 0 | NA |
| 127 | emerge | 4 | female | 106121432 | 106458216 | 336785 | 0 | NA |
| 128 | emerge | 10 | male | 47013328 | 47167032 | 153705 | 0 | NA |
| 129 | emerge | 22 | female | 15447504 | 49498590 | 34051087 | 0 | NA |
| 130 | emerge | 16 | female | 37354 | 2085788 | 2048435 | 0 | NA |
| 131 | emerge | 2 | male | 24096631 | 26806502 | 2709872 | 0 | NA |
| 131 | emerge | 3 | male | 40737026 | 42627678 | 1890653 | 0 | NA |
| 132 | emerge | 4 | female | 56015824 | 60853243 | 4837420 | 0 | NA |
| 133 | emerge | 4 | male | 105914711 | 106554939 | 640229 | 1 | MYLEODYS-PLASTIC SYNDROME |
| 134 | emerge | 9 | male | 36587 | 1653954 | 1617368 | 0 | NA |
| 135 | emerge | 7 | male | 89913562 | 149214103 | 59300542 | 0 | NA |
| 136 | emerge | 4 | male | 105748162 | 106234442 | 486281 | 0 | NA |
| 137 | emerge | 13 | female | 49508271 | 50336803 | 828533 | 0 | NA |
| 138 | emerge | 20 | male | 35931058 | 45036045 | 9104988 | 0 | NA |
| 139 | emerge | 15 | female | 53362172 | 100198883 | 46836712 | 0 | NA |
| 140 | emerge | 8 | female | 166818 | 146174216 | 146007398 | 1 | NON-HODGKIN LYMPHOMA |
| 141 | emerge | 4 | female | 53542981 | 190993476 | 137450496 | 0 | NA |
| 142 | emerge | 17 | male | 41544850 | 42439278 | 894429 | 0 | NA |
| 143 | emerge | 8 | male | 229299 | 146142241 | 145912943 | 0 | NA |
| 144 | emerge | 16 | male | 46479495 | 88640608 | 42161114 | 0 | NA |
| 145 | emerge | 4 | male | 105784667 | 107173741 | 1389075 | 0 | NA |
| 146 | emerge | 4 | male | 53338236 | 191164126 | 137825891 | 1 | LEUKEMIA |
| 147 | emerge | 3 | female | 85634043 | 87010264 | 1376222 | 0 | NA |
| 148 | emerge | 7 | female | 141726987 | 142031222 | 304236 | 0 | NA |
| 149 | emerge | 9 | female | 195964 | 33508281 | 33312318 | 0 | NA |
| 150 | emerge | 13 | male | 28954562 | 66694128 | 37739567 | 0 | NA |
| 151 | emerge | 17 | female | 53011 | 78599575 | 78546564 | 0 | NA |
| 152 | emerge | 7 | male | 105208804 | 149182926 | 43974123 | 0 | NA |
| 153 | emerge | 8 | female | 117892398 | 118027736 | 135339 | 0 | NA |
| 154 | emerge | 4 | male | 209538 | 7076810 | 6867273 | 0 | NA |
| 155 | emerge | 17 | male | 51088 | 20852502 | 20801415 | 0 | NA |
| 156 | emerge | 4 | male | 105897609 | 107766967 | 1869359 | 1 | MULTIPLE MYELOMA |
| 157 | emerge | 14 | female | 20876340 | 106347672 | 85471333 | 0 | NA |
| 158 | emerge | 9 | male | 256505 | 33170228 | 32913724 | 1 | LEUKEMIA |
| 159 | emerge | 15 | female | 18421386 | 99980176 | 81558791 | 0 | NA |
| 160 | emerge | 14 | female | 46966619 | 106345097 | 59378479 | 0 | NA |
| 161 | emerge | 11 | female | 106764075 | 126497931 | 19733857 | 0 | NA |
| 162 | emerge | 13 | female | 17868000 | 113905078 | 96037078 | 0 | NA |
| 163 | emerge | 2 | female | 25007490 | 26134743 | 1127254 | 0 | NA |
| 164 | emerge | 6 | male | 134113 | 26256979 | 26122867 | 0 | NA |
| 165 | emerge | 22 | male | 21978009 | 49518559 | 27540551 | 0 | NA |
| 166 | emerge | 14 | male | 19335041 | 106358708 | 87023668 | 0 | NA |
| 167 | emerge | 20 | male | 14832301 | 14989361 | 157061 | 0 | NA |
| 168 | emerge | 11 | female | 63805488 | 134444057 | 70638570 | 0 | NA |
| 169 | emerge | 11 | male | 107128834 | 111229343 | 4100510 | 0 | NA |
| 2 | garnet | 20 | female | 31351079 | 47265311 | 15914233 | 0 | NA |
| 4 | garnet | 20 | female | 34660287 | 43174937 | 8514651 | 0 | NA |
| 7 | garnet | 1 | female | 229535576 | 229875008 | 339433 | 1 | NON-HODGKIN LYMPHOMA |
| 8 | garnet | 15 | female | 28723577 | 29168271 | 444695 | 0 | NA |
| 10 | garnet | 4 | female | 103608433 | 191000331 | 87391899 | 0 | NA |
| 12 | garnet | 4 | female | 105982514 | 106608151 | 625638 | 0 | NA |
| 13 | garnet | 2 | female | 40814 | 74951691 | 74910878 | 0 | NA |
| 15 | garnet | 7 | female | 37568071 | 39958198 | 2390128 | 0 | NA |
| 17 | garnet | 14 | female | 71746253 | 106358708 | 34612456 | 0 | NA |
| 20 | garnet | 6 | female | 29826769 | 34226564 | 4399796 | 0 | NA |
| 21 | garnet | 17 | female | 53206 | 7885267 | 7832062 | 0 | NA |
| 22 | garnet | 16 | female | 76654019 | 84124657 | 7470639 | 0 | NA |
| 23 | garnet | 11 | female | 111590526 | 115287675 | 3697150 | 0 | NA |
| 27 | garnet | 4 | female | 65174 | 3774084 | 3708911 | 0 | NA |
| 28 | garnet | 16 | female | 28918272 | 31405522 | 2487251 | 0 | NA |
| 30 | garnet | 7 | female | 122781030 | 135633492 | 12852463 | 1 | NON-HODGKIN LYMPHOMA |
| 30 | garnet | 11 | female | 109755301 | 116061136 | 6305836 | 1 | NON-HODGKIN LYMPHOMA |
| 31 | garnet | 7 | female | 100353939 | 101606035 | 1252097 | 1 | LEUKEMIA |
| 32 | garnet | 19 | female | 58541258 | 58722855 | 181598 | 0 | NA |
| 33 | garnet | 17 | female | 26040058 | 27404494 | 1364437 | 0 | NA |
| 35 | garnet | 14 | female | 23062136 | 104708792 | 81646657 | 0 | NA |
| 36 | garnet | 7 | female | 4149376 | 4230185 | 80810 | 0 | NA |
| 37 | garnet | 14 | female | 53831209 | 106345097 | 52513889 | 0 | NA |
| 38 | garnet | 10 | female | 55541277 | 135054999 | 79513723 | 0 | NA |
| 38 | garnet | 11 | female | 79429411 | 114388728 | 34959318 | 0 | NA |
| 39 | garnet | 2 | female | 85808055 | 88906246 | 3098192 | 0 | NA |
| 39 | garnet | 12 | female | 61880 | 132287718 | 132225839 | 0 | NA |
| 39 | garnet | 19 | female | 211912 | 63785276 | 63573365 | 0 | NA |
| 40 | garnet | 5 | female | 156581710 | 157125213 | 543504 | 1 | LEUKEMIA |
| 40 | garnet | 5 | female | 63863265 | 64906443 | 1043179 | 1 | LEUKEMIA |
| 40 | garnet | 5 | female | 123826877 | 124116778 | 289902 | 1 | LEUKEMIA |
| 40 | garnet | 13 | female | 19134385 | 113948457 | 94814073 | 1 | LEUKEMIA |
| 41 | garnet | 22 | female | 16545995 | 49528625 | 32982631 | 0 | NA |
| 43 | garnet | 8 | female | 142506534 | 146255887 | 3749354 | 0 | NA |
| 44 | garnet | 1 | female | 72056794 | 100827590 | 28770797 | 0 | NA |
| 44 | garnet | 1 | female | 788822 | 71939004 | 71150183 | 0 | NA |
| 45 | garnet | 2 | female | 25301554 | 25408022 | 106469 | 0 | NA |
| 46 | garnet | 11 | female | 181956 | 20329365 | 20147410 | 0 | NA |
| 47 | garnet | 6 | female | 63688718 | 82913128 | 19224411 | 0 | NA |
| 48 | garnet | 17 | female | 26164312 | 27254015 | 1089704 | 0 | NA |
| 51 | garnet | 20 | female | 45541548 | 62382907 | 16841360 | 0 | NA |
| 52 | garnet | 1 | female | 758311 | 19071808 | 18313498 | 0 | NA |
| 53 | garnet | 8 | female | 91821032 | 99032993 | 7211962 | 0 | NA |
| 54 | garnet | 4 | female | 91700294 | 191176659 | 99476366 | 0 | NA |
| 55 | garnet | 11 | female | 194062 | 47793909 | 47599848 | 0 | NA |
| 58 | garnet | 5 | female | 91093297 | 107770468 | 16677172 | 0 | NA |
| 60 | garnet | 11 | female | 71532141 | 134443680 | 62911540 | 0 | NA |
| 64 | garnet | 20 | female | 30648260 | 48487515 | 17839256 | 0 | NA |
| 65 | garnet | 2 | female | 40814 | 919469 | 878656 | 0 | NA |
| 66 | garnet | 14 | female | 66034953 | 106292845 | 40257893 | 0 | NA |
| 67 | garnet | 15 | female | 18436314 | 100214840 | 81778527 | 0 | NA |
| 69 | garnet | 13 | female | 49622056 | 50501769 | 879714 | 0 | NA |
| 72 | garnet | 1 | female | 863421 | 120236149 | 119372728 | 0 | NA |
| 74 | garnet | 22 | female | 16163945 | 49518363 | 33354419 | 0 | NA |
| 75 | garnet | 8 | female | 253427 | 146221527 | 145968101 | 0 | NA |
| 76 | garnet | 19 | female | 34777847 | 44227556 | 9449710 | 0 | NA |
| 76 | garnet | 19 | female | 211912 | 3173856 | 2961945 | 0 | NA |
| 76 | garnet | 19 | female | 6213355 | 7854890 | 1641536 | 0 | NA |
| 77 | garnet | 2 | female | 2705540 | 70866242 | 68160703 | 0 | NA |
| 79 | garnet | 15 | female | 18436314 | 100216154 | 81779841 | 0 | NA |
| 82 | garnet | 7 | female | 109042895 | 146228703 | 37185809 | 0 | NA |
| 83 | garnet | 16 | female | 82765533 | 87444435 | 4678903 | 0 | NA |
| 83 | garnet | 16 | female | 28733106 | 31220356 | 2487251 | 0 | NA |
| 84 | garnet | 17 | female | 26040058 | 27185863 | 1145806 | 0 | NA |
| 85 | garnet | 10 | female | 34530860 | 34584886 | 54027 | 0 | NA |
| 89 | garnet | 9 | female | 11112042 | 11364073 | 252032 | 0 | NA |
| 90 | garnet | 20 | female | 30355615 | 44073632 | 13718018 | 0 | NA |
| 91 | garnet | 4 | female | 190308979 | 190464285 | 155307 | 0 | NA |
| 91 | garnet | 8 | female | 172340 | 255337 | 82998 | 0 | NA |
| 91 | garnet | 16 | female | 26671 | 262935 | 236265 | 0 | NA |
| 93 | garnet | 15 | female | 70761700 | 73307998 | 2546299 | 0 | NA |
| 94 | garnet | 2 | female | 40814 | 88172858 | 88132045 | 0 | NA |
| 95 | garnet | 17 | female | 26164312 | 27196402 | 1032091 | 0 | NA |
| 98 | garnet | 9 | female | 36587 | 28238076 | 28201490 | 0 | NA |
| 99 | garnet | 2 | female | 23019681 | 25942273 | 2922593 | 0 | NA |
| 99 | garnet | 10 | female | 134008874 | 134256391 | 247518 | 0 | NA |
| 100 | garnet | 7 | female | 64579322 | 158811981 | 94232659 | 1 | LEUKEMIA |
| 101 | garnet | 4 | female | 106364184 | 106723939 | 359756 | 0 | NA |
| 103 | garnet | 11 | female | 57307361 | 134404931 | 77097570 | 0 | NA |
| 104 | garnet | 7 | female | 100399198 | 100914175 | 514978 | 0 | NA |
| 106 | garnet | 13 | female | 49522141 | 50460373 | 938233 | 0 | NA |
| 107 | garnet | 1 | female | 211718771 | 227118755 | 15399985 | 0 | NA |
| 107 | garnet | 12 | female | 61880 | 132287718 | 132225839 | 0 | NA |
| 111 | garnet | 3 | female | 118218683 | 118453038 | 234356 | 0 | NA |
| 113 | garnet | 11 | female | 63906946 | 134405679 | 70498734 | 0 | NA |
| 114 | garnet | 2 | female | 231979528 | 242366419 | 10386892 | 0 | NA |
| 3 | gecco2 | 9 | female | 36587 | 38446840 | 38410254 | 0 | NA |
| 56 | gecco2 | 1 | female | 229271417 | 247146479 | 17875063 | 0 | NA |
| 59 | gecco2 | 2 | female | 56451813 | 56824236 | 372424 | 0 | NA |
| 63 | gecco2 | 2 | female | 10325386 | 26586236 | 16260851 | 0 | NA |
| 1 | hpfx550 | 9 | female | 36587 | 38761831 | 38725244 | 0 | NA |
| 5 | hpfx550 | 9 | female | 545293 | 5453988 | 4908696 | 0 | NA |
| 6 | hpfx550 | 8 | female | 14660345 | 14884216 | 223872 | 0 | NA |
| 9 | hpfx550 | 7 | female | 82025167 | 102849653 | 20824487 | 0 | NA |
| 11 | hpfx550 | 8 | female | 14089622 | 26964516 | 12874895 | 0 | NA |
| 14 | hpfx550 | 9 | female | 18762370 | 19416464 | 654095 | 0 | NA |
| 16 | hpfx550 | 10 | female | 27318632 | 61073946 | 33755315 | 0 | NA |
| 18 | hpfx550 | 6 | female | 110391 | 28122576 | 28012186 | 1 | NON-HODGKIN LYMPHOMA |
| 19 | hpfx550 | 11 | female | 61337211 | 93050801 | 31713591 | 0 | NA |
| 24 | hpfx550 | 20 | female | 32027739 | 62349775 | 30322037 | 0 | NA |
| 25 | hpfx550 | 13 | female | 77492042 | 89493256 | 12001215 | 0 | NA |
| 26 | hpfx550 | 8 | female | 27933928 | 80036490 | 52102563 | 0 | NA |
| 26 | hpfx550 | 11 | female | 62361643 | 134321343 | 71959701 | 0 | NA |
| 26 | hpfx550 | 18 | female | 42389048 | 54819490 | 12430443 | 0 | NA |
| 29 | hpfx550 | 3 | female | 161200962 | 161597698 | 396737 | 0 | NA |
| 34 | hpfx550 | 9 | female | 85443302 | 140131592 | 54688291 | 0 | NA |
| 42 | hpfx550 | 3 | female | 60928629 | 75610832 | 14682204 | 0 | NA |
| 49 | hpfx550 | 1 | female | 995669 | 100089177 | 99093509 | 0 | NA |
| 50 | hpfx550 | 13 | female | 44267779 | 53595673 | 9327895 | 1 | LEUKEMIA |
| 57 | hpfx550 | 13 | female | 105087376 | 105281430 | 194055 | 0 | NA |
| 61 | hpfx550 | 10 | female | 47058066 | 47959596 | 901531 | 0 | NA |
| 62 | hpfx550 | 8 | female | 102031086 | 146174216 | 44143131 | 0 | NA |
| 68 | hpfx550 | 3 | female | 197222803 | 197458578 | 235776 | 0 | NA |
| 70 | hpfx550 | 13 | female | 47220476 | 59116317 | 11895842 | 1 | NON-HODGKIN LYMPHOMA |
| 71 | hpfx550 | 15 | female | 54853326 | 55563118 | 709793 | 0 | NA |
| 73 | hpfx550 | 22 | female | 20729916 | 21538519 | 808604 | 0 | NA |
| 78 | hpfx550 | 3 | female | 21979879 | 22134900 | 155022 | 0 | NA |
| 80 | hpfx550 | 8 | female | 70489772 | 71109183 | 619412 | 0 | NA |
| 81 | hpfx550 | 13 | female | 45996807 | 113927626 | 67930820 | 0 | NA |
| 86 | hpfx550 | 6 | female | 134113 | 26433867 | 26299755 | 0 | NA |
| 87 | hpfx550 | 10 | female | 135116379 | 135284293 | 167915 | 0 | NA |
| 88 | hpfx550 | 9 | female | 118493276 | 118566196 | 72921 | 0 | NA |
| 92 | hpfx550 | 12 | female | 13083502 | 37884616 | 24801115 | 0 | NA |
| 96 | hpfx550 | 4 | female | 106086173 | 106698459 | 612287 | 0 | NA |
| 97 | hpfx550 | 1 | female | 77672228 | 121236957 | 43564729 | 0 | NA |
| 97 | hpfx550 | 7 | female | 73481452 | 158612946 | 85131495 | 0 | NA |
| 102 | hpfx550 | 4 | female | 82103746 | 190927939 | 108824194 | 0 | NA |
| 105 | hpfx550 | 17 | female | 26034031 | 27338617 | 1304587 | 0 | NA |
| 108 | hpfx550 | 7 | female | 35960726 | 47737034 | 11776309 | 0 | NA |
| 109 | hpfx550 | 5 | female | 84447374 | 160469603 | 76022230 | 0 | NA |
| 109 | hpfx550 | 20 | female | 34317738 | 49606347 | 15288610 | 0 | NA |
| 110 | hpfx550 | 13 | female | 46325627 | 51370869 | 5045243 | 1 | LEUKEMIA |
| 112 | hpfx550 | 10 | female | 53510977 | 53657830 | 146854 | 0 | NA |

**Table S4.** Correlation of case status with hematologic cancer and mosaic anomaly status.

| **Cohort** | **Outcome** | **Cases: Controls** | **Previous Case status~mosaic anomaly** | | | **Previous Case status~hematologic cancer** | | |
| --- | --- | --- | --- | --- | --- | --- | --- | --- |
|  |  |  | **Correlation** | **95% CI** | **P-value** | **Correlation** | **95% CI** | **P-value** |
| eMERGE | Dementia | 786:1571 | -0.008 | -0.05 to 0.03 | 0.7 | -0.05 | -0.09 to -0.005 | 0.03 |
| Hip Fracture | Hip fractures | 2069:2385 | -0.02 | 0.05 to 0.009 | 0.2 | 0.02 | -0.1 to 0.05 | 0.3 |
| GECCO | Colorectal cancer | 431:427 | 0.06 | -0.008 to 013 | 0.08 | 0.009 | -0.06 to 0.08 | 0.8 |
| GARNET | Metabolic/CVD | 2246:2261 | -0.003 | -0.03 to 0.03 | 0.8 | 0.01 | -0.02 to 0.04 | 0.4 |

**Table S5.** Counts and frequencies of detectable chromosomal mosaicism with case status.

| Study: Case or Control Status | Non-Mosaic (n) | Mosaic (n) | Non-Mosaic (%) | Mosaic (%) |
| --- | --- | --- | --- | --- |
| eMERGE: Alzheimer case | 769 | 17 | 97.84 | 2.16 |
| eMERGE: Control | 1533 | 38 | 97.58 | 2.42 |
| GECCO: Colorectal cancer case | 428 | 3 | 99.30 | 0.70 |
| GECCO: Control | 426 | 1 | 99.77 | 0.23 |
| GARNET: Metabolic/CVD case | 2212 | 34 | 98.49 | 1.51 |
| GARNET: Control | 2224 | 37 | 98.36 | 1.64 |
| Hip Fracture: Hip facture case | 2057 | 12 | 99.42 | 0.58 |
| Hip Fracture: Control | 2358 | 27 | 98.87 | 1.13 |
| All studies: Hematologic cancer case | 213 | 16 | 93.01 | 6.99 |
| All Studies: Control | 11794 | 153 | 98.72 | 1.28 |
